# A novel proficiency test to assess the animal diagnostic investigation process in identifying an unknown toxicant

**DOI:** 10.1016/j.toxrep.2025.101925

**Published:** 2025-01-26

**Authors:** Andriy Tkachenko, Yang Chen, Marissa Petrey, Scott Fritz, Tim Walsh, David Rotstein, Megan R. Miller, Bruce Williams, Michael Dark, Matthew Kmet, Ravinder Reddy, Gregory Tyson, Sarah M. Nemser

**Affiliations:** a US Food and Drug Administration, Center for Veterinary Medicine, Laurel, MD 20708, USA; b US Food and Drug Administration, Human Foods Program, Bedford Park, IL 60501, USA; c Institute for Food Safety and Health, Bedford Park, Illinois, IL 60501, USA; d Kansas State University, Veterinary Diagnostic Laboratory, Manhattan, KS 66506, USA; e Davis-Thompson Foundation, Gurnee, IL 60031, USA

**Keywords:** Proficiency testing, Assessment of performance, Lead toxicosis, Animal diagnostic, Investigation process

## Abstract

Participation in Proficiency Tests (PTs) is an important component of quality assurance in testing laboratories. In a typical chemistry PT, blind-coded samples are sent to participants for analysis of specific chemical agents, and results are compared to a pre-determined key (e.g., expected concentrations) to assess proficiency. In the animal diagnostic PT presented here, organizers evaluated not only the analytical component of the diagnostic investigation but also the entire investigative process as a multi-step, holistic multidisciplinary approach. Fourteen veterinary diagnostic laboratories (VDLs) participated in an exercise to identify the root cause of a simulated case of lead (Pb) toxicosis. VDLs received a case description outlining neurological signs in cattle, a digitized brain histology slide, and liver and brain tissue samples for optional chemistry analysis. Thirteen of 14 VDLs successfully diagnosed lead toxicosis by completing the following stages: (a) correctly identifying histological abnormalities, (b) providing three adequate differential diagnoses, (c) selecting adequate chemistry analyses to rule in or rule out possible causes, (d) accurately detecting lead concentration in the liver, and (e) interpreting the diagnostic significance of their results correctly. Importantly, participants first had to determine which chemistry analyses were appropriate and then to accurately quantify the target analytes. This approach provided greater confidence in the diagnostic capability of the laboratory by reducing the bias associated with being given a known chemical contaminant for which to test, typical of most chemistry PTs, and may therefore be of interest to PT providers and accreditation committees.

## Highlights

• Typical proficiency tests focus on the analytical part of diagnostic investigation.
• The novel proficiency test evaluates the entire animal diagnostic investigation.
• The novel approach mimics real world toxicosis cases.
• The novel approach provides unbiased evaluation of toxicosis investigations.

## 1 Introduction

**Fig. 1:**
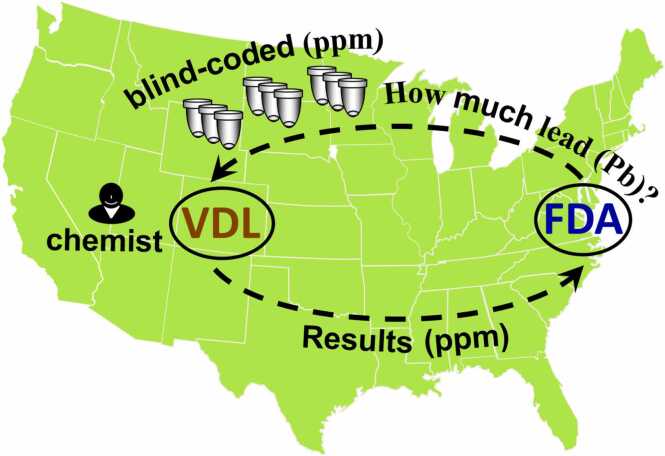
Typical chemistry PT conducted by FDA-Vet-LIRN focusing on the analytical part of the diagnostic investigation process in public veterinary diagnostic laboratories (VDLs).

The U.S. Food and Drug Administration’s (FDA) Center for Veterinary Medicine (CVM) investigates animal health issues potentially linked to CVM-regulated products, such as animal foods, feeds, and drugs. The FDA’s Veterinary Laboratory Investigation and Response Network (Vet-LIRN) [1] consists of a collaborative partnership between CVM and 47 veterinary diagnostic laboratories (VDLs), which are mostly located in academia and assist CVM investigations by testing samples for microbial pathogens and chemical agents including pesticides, mycotoxins, metals, vitamins, and others. Unlike FDA laboratories, VDLs can test animal specimens and their testing results can support regulatory activities by CVM and the Office of Investigations and Inspections. Specifically, findings from Vet-LIRN laboratory testing can guide regulatory sampling and testing by FDA regulatory laboratories. Given the importance of Vet-LIRN laboratory sample results, Vet-LIRN regularly conducts both microbiology and chemistry Proficiency Tests (PTs) to assess the analytical competency of member laboratories [2]. A typical chemistry PT involves preparing and shipping blind-coded samples to laboratories for analysis of specific chemicals (Fig. 1). The results are then compared to a pre-determined key (e.g., expected chemical concentrations) to evaluate the proficiency of participants. Participation in PTs and similar external quality assessment (EQA) exercises is an important component of quality assurance in testing laboratories, and results are used to obtain and maintain accreditations [3].

Separate from Vet-LIRN, VDLs conduct investigations of animal health issues from a variety of sources, including farmers, pet owners, zoos, veterinary clinics, and wildlife organizations. By investigating animal illnesses and animal foods that may have contributed to those illnesses, VDL investigations also serve as an early warning system for potential issues with human foods that are similarly sourced. This role was demonstrated during the melamine adulteration events. In 2007, melamine was identified in U.S. pet food, leading the FDA to take proactive measures to ensure the safety of human food [4]. This contrasted with China, where 294,000 infants were poisoned by melamine-adulterated milk products in 2008 according to the Chinese Ministry of Health [5], [6]. More recently, in 2020, two VDLs found high concentrations of aflatoxins in a widely distributed commercial dog food linked to several cases of hepatic failure in dogs [7], prompting the manufacturer to voluntarily recall the contaminated dog foods [8], which were made with aflatoxin contaminated corn. The early detection by these VDLs not only prevented harm to animals but may have also helped prevent the contaminated corn from entering the human food supply, further supporting the importance of VDLs in protecting human food safety as part of a One Health approach.

Animal toxicosis investigations usually follow an interdisciplinary (e.g., veterinary medicine, pathology, analytical chemistry, and toxicology) systematic search for unknown toxicants, where each subsequent step is determined by the outcome of the preceding one [9]. The process typically begins by evaluating the case report, which may include the animal’s clinical signs, dietary and medical history, a description of the exposure site, exposure duration and surrounding circumstances, and any additional evidence and clues of what may have occurred. This is followed by determining relevant ancillary tests and procedures (e.g., necropsy, biopsy, histology, blood work, screening for pathogens and others) which are conducted in-house or requested from subcontracted laboratories. The results obtained may not be sufficient for a definitive diagnosis and are typically used to generate differential diagnoses needed for the next step, such as selecting a shortlist of chemical analyses to be performed on animal specimens. This systematic narrowing down of possibilities is crucial in any investigation, as testing for *all* possible contaminants is impractical due to limited resources and sample availability [9]. Chemical analysis is usually performed by an analytical chemist and the results are interpreted by a toxicologist requiring expertise in diagnostic levels of concern for various contaminants. The toxicology findings are then integrated with other data to complete the final step of the investigative process: correctly interpreting all the data and, ideally, arriving at an accurate diagnosis that identifies the toxicant and its effects on the animal.

This manuscript reports a PT designed to evaluate not only the analytical component of the diagnostic investigation but also the entire investigative process as a multi-step, holistic approach requiring integration of veterinary medicine, pathology, analytical chemistry, and toxicology efforts.

## 2 Materials and methods

### 2.1 Design of the diagnostic PT

**Fig. 2:**
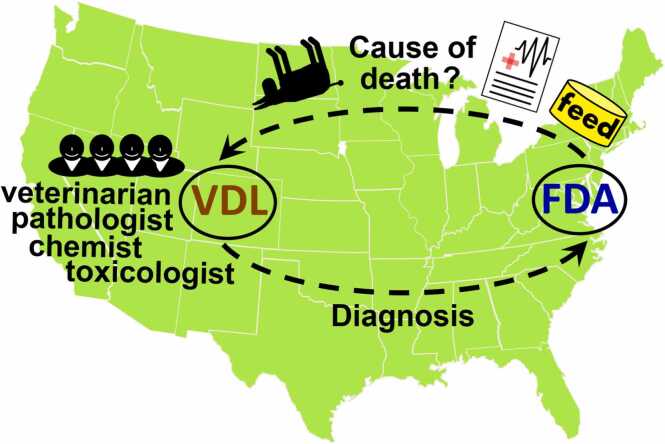
Schematic presentation of the diagnostic PT evaluating the investigative process as a holistic approach based on veterinary medicine, pathology, analytical chemistry, and toxicology assessments.

In the animal diagnostic PT described (Fig. 2), each participating laboratory was tasked with identifying the root cause of simulated case of lead (Pb) toxicosis requiring a systematic step-by-step investigation using an integrative, multidisciplinary approach. In the first stage, participants had to evaluate the case for evidence and clues needed to successfully assess the histopathology data and generate three adequate differential diagnoses. Importantly, in the next stage, participants had to determine which chemistry tests are needed to be performed on the PT samples to rule in or rule out the causes of illness. The applied analytical tests had to be accurate enough and participants had to correctly interpret the diagnostic significance of the analytical results. Ultimately participants needed to combine all the data to reach the final diagnosis of lead toxicosis.

### 2.2 PT items and procedure

**Fig. 3:**
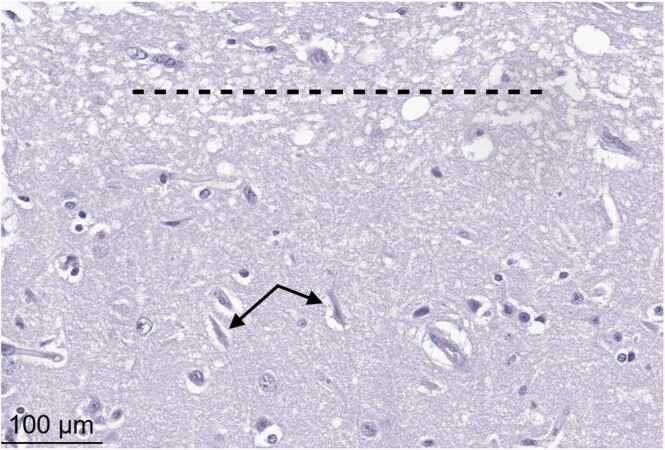
The digitized slide (bovine brain, HE 200X) provided to 14 participating laboratories via e-portal [10]. All 14 participating laboratories noted the neuronal necrosis (arrows are indicated here for clarity but not during the PT) and edema and pallor of the white matter (malacia; dotted line is indicated here for clarity but not during the PT).

FDA-Vet-LIRN extended an invitation through the e-portal to its network laboratories to participate and fourteen laboratories agreed to join the PT. Each participant received: (i) a case describing cattle with neurological signs (Table 1), (ii) a website link [10] to a digitized histology slide of brain showing morphological abnormalities of cerebrocortical necrosis and malacia (Fig. 3), and (iii) frozen brain and liver specimens for possible chemistry analyses, which had to be determined by participants based on (i) and (ii). Two FAQ teleconference sessions were offered to participants prior to the PT sample shipping date.

**Table 1:** The case description received by 14 participating VDLs.

*“A herd of 80 head of crossbred Angus cow-calf pairs experienced multiple calf deaths in the last week. The herd is currently pastured on 250 acres of native grass that is used every summer for grazing the herd. There is one big pond that shrunk by about 50 % over the last month as well as a seasonal creek that is dry. All but one of the calves were found dead several days post-mortem based upon the condition of the carcass and extensive scavenging. The owner started checking them more regularly and noticed a clinical animal yesterday. The calf in question was in a small group of trees with no other animals around. The calf could not seem to find its way out of the trees and ran into several of them. The calf appeared to be ataxic, the owner saw the calf lay down, start to seize, and die. Clinical signs included: ataxia, seizure and death. Diet: native grass and Creep feed. Submitted samples include brain, liver, water, and a sample of a Creep feed. The total dietary sulfur content was estimated to be 0.28 % with the submitted feed. Available diagnostic samples: unknown brain and unknown liver. QC samples (“normal” liver and brain) are also provided. Digitized histology image of the unknown brain is available via this weblink: …”*

Participants were asked to review the case description and histology data provided and to report the following within 14 days: their histopathologic findings, the top three differential diagnoses, and a decision-making summary. Participants were asked to report histopathology evaluations before the chemistry testing to ensure that the analytical results don’t impact the three differential diagnoses. The following were due within 21 days after the sample shipment: a list of chemistry analyses chosen to be performed, a description of the instrumentation used, the expected sensitivities of the methods used, results (e.g., concentrations of analytes) from the chemistry analyses, an interpretation of the chemistry results based on expertise regarding diagnostic levels of concern for contaminants, and the final diagnosis requiring interpretation of all data combined. All communication and data submissions were conducted via the e-portal.

### 2.3 Preparation and verification of PT tissue samples

Each participant received four tissue samples for optional chemistry analysis: an unknown brain sample (the cattle case), a known normal brain sample for optional QA/QC analysis, an unknown liver sample (the cattle case), and a known normal liver sample for optional QA/QC analysis. All four tissue portions were collected from healthy bovines (containing only trace amounts of lead). The unknown liver sample (the cattle case) was prepared by fortifying 200 g of the normal (negative control) liver with lead at a concentration of 16 µg/g wet weight. To ensure consistency both among participants and within each sample portion, all four tissue portions were pre-homogenized by the organizers. The assigned value (i.e., the best estimate of the true concentration of lead) and sample homogeneity were established through analysis using a previously reported method [11]. Three PT samples (Table 4) were randomly selected and analyzed in duplicate for the fortified liver, while single analyses were conducted for three unfortified (normal) liver samples. The PT liver samples were confirmed to be sufficiently homogeneous in accordance with ISO/IEC 13528:2022 [12].

## 3 Results

### 3.1 Reporting

Frozen brain and liver samples were shipped using overnight delivery services, and all participants confirmed receipt the following day. All 14 participants submitted their histopathological evaluations and three differential diagnoses within 14 days as requested. All 14 participants reported their chemistry analysis results (concentration of analytes) and their diagnostic significance within 21 days as requested. Lab ID #4 encountered technical problems with the chemical analysis of samples and subcontracted the lead testing to another laboratory.

### 3.2 Performance of participants in the diagnostic PT

**Fig. 4:**
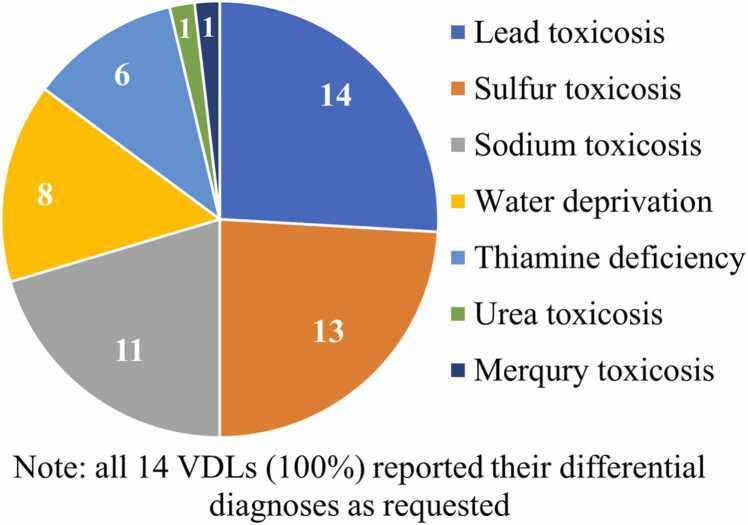
Summary of differential diagnoses submitted by all PT participants.

A total of 93 % (13/14) of the participating VDLs correctly concluded the diagnosis (lead toxicosis) and successfully completed the following seven steps of the diagnostic PT: (1) Submitted their histopathologic evaluations, which varied in the degree of detail (not shown); (2) Correctly identified histological abnormalities of cerebrocortical necrosis and/or polioencephalomalacia, which can be associated with various causes; (3) Suggested an adequate list of top three differential diagnoses (Table 2 and Fig. 4) based on the case description and the histopathology findings; (4) Selected adequate chemistry analyses (Table 3) for brain and liver samples; (5) Accurately detected lead in the bovine liver (e.g., labs reported lead at 11–17 µg/g, demonstrating the satisfactory accuracy of their chemistry methods for the expected value of 16 µg/g); (6) Correctly interpreted diagnostic significance of their chemistry results based on a threshold of 5 µg/g in wet liver. It is important to note that this 5 µg/g value is not an exact regulatory limit but rather an approximate guideline based on expert opinion among toxicologists, who generally consider lead concentrations above this level to be of concern. Importantly, the information regarding the level of concern for lead in bovine liver (5 µg/g) was not provided to participating VDLs during this PT. The PT organizers expected the VDLs to rely on their expertise and knowledge of diagnostically relevant levels of concern for contaminants, including lead, to correctly interpret their test results; and (7) Integrated their toxicology findings with other data to correctly conclude the diagnosis of lead intoxication.

**Table 2:** Differential diagnoses submitted by each participating laboratory^‡^.

Lab ID#	Lead toxicosis			Sulfur toxicosis			Sodium toxicosis			Water deprivation			Thiamine deficiency			Other toxicosis
	1st	2nd	3rd	1st	2nd	3rd	1st	2nd	3rd	1st	2nd	3rd	1st	2nd	3rd	3rd
1			1	1				1			1		1			
2			1		1		1			1				1		
3	1					1					1					
4			1	1				1			1			1		
5	1					1		1							1	
6	1					1		1								
7	1				1				1			1		1		
8		1				1	1			1						
9	1					1		1			1					
10			1	1				1								
11	1							1			1					Urea
12	1				1										1	
13	1				1				1							
14		1		1												Hg
Total	8	2	4	4	4	5	2	7	2	2	5	1	1	3	2	2

^‡^Note, these differential diagnoses means that VDLs short-listed potential causes of toxicosis allowing them to narrow down the list of potential chemicals to be tested for (see Table 3) in liver and brain specimens in the next step of the diagnostic investigation.

**Table 3:** Chemistry analyses performed by participating VDLs.

Lab ID #	Analyte tested	Unknown brain (µg/g)	Unknown liver (µg/g)	LOD/LOQ (µg/g)	Instruments used
1	**Lead**	0.17 (dry weight)	55 (dry weight)*	0.01	ICP-MS
2	Sodium	1600	1383	125/250	ICP-OES
	**Lead**	NT	15*	0.05/0.1	ICP-MS
3	Sodium	1505	NT	NR	ICP-MS
	**Lead**	NT	14*	0.5	ICP-OES
4	Sulfur	NR	NT	NR	NR
	**Lead**	NT	0.24 (dry weight)**	NR	NR
5	Sodium	1908	NT	0.1	ICP-MS ICP-MS
	**Lead**	NT	**66 (dry weight**)*	25	
6	Sodium	1960	NT	35	ICP-MS ICP-MS
	**Lead**	NT	16*	0.05	
7	**Lead**	NT	16*	0.02	ICP-MS
8	Sodium	1800	NT	4	ICP-OES
	**Lead**	NT	14*	0.005	ICP-MS
9	Sodium	1800	NT	3	ICP-OES
	**Lead**	NT	15*	1	ICP-MS
	Other elements	Sulfur 1200	Unremarkable	0.3	ICP-MS
10	Sodium	2240^†^	NT	200	FAAS
	**Lead**	NT	11*	0.1	FAAS
11	Sodium	1721	NT	NR	ICP-MS
	Lead	NT	14*	0.1	ICP-MS
	Other metals	NT	Unremarkable	0.01–0.2	ICP-MS
12	**Lead**	1.06**	16*	0.1	ICP-OES
13	**Lead**	NT	13*	0.02	ICP-MS
14	**Lead**	NT	13*	0.005	ICP-MS

LOD = limit of detection; LOQ = limit of quantification; NT = not tested; NR = not reported; ICP-MS = inductively coupled plasma mass spectrometry; ICP-OES = inductively coupled plasma optical emission spectroscopy. FAAS = Flame Atomic Absorption Spectrometry. *Lead concentration meets PT organizers’ expectations. **Lead concentration does not meet organizers’ expectations.

**Table 4:** Preparation and Verification of PT liver samples by PT organizers.

Sample #	Normal liver (negative control, unfortified, µg/g)	Unknown liver (the cattle case) prepared by fortification		
		Analysis results (µg/g) for portion “A”	Analysis results (µg/g) for portion “B”	Target concentration (µg/g) by fortification with lead
1	0.016	16.53	16.39	15
2	0.011	16.35	16.05	
3	0.014	15.89	16.87	
Mean	0.014	16.35*		n/a
RSD (%)	18.4 %	2.1 %**		n/a

* The assigned value (i.e., the best estimate of the true concentration of lead).

** Concluded as sufficiently homogeneous.

One laboratory (Lab ID #4) successfully completed steps 1, 2, 3, 4, and 6 of the PT. However, in step 5, their testing revealed only 0.24 µg/g of Pb, causing them to mistakenly rule out lead toxicosis in step 7. Lab ID #4 reported an unsatisfactory low value, which was obtained by an outsourced laboratory and was requested to perform a root cause analysis as a follow-up on the PT.

### 3.3 Conclusions on the performance of participants in identifying the lead toxicosis

All but one laboratory, which reported an unsatisfactory underestimated value, successfully identified lead toxicosis as the cause of the animal’s illness, regardless of the methods used. The PT results provide a high degree of confidence that VDLs will deliver reliable data in future cases involving lead exposure. This is a significant finding because lead exposure in animals, including livestock, wildlife, and pets, is common due to the ingestion of lead from sources such as shotgun pellets, fishing sinkers, paint, improperly discarded batteries, and contaminated water and soil near mining and smelting sites, which can also contaminate animal food products.

## 4 Discussion

### 4.1 Comparison of the diagnostic PT conducted versus similar exercises by others

Previously reported PTs and EQAs by others vary in their designs and schemes, which depend greatly on the goals of each exercise [2], [13]. Most PTs focus on evaluating a specific diagnostic component, such as pre-analytical (e.g., review of clinical history and evidence, sample handling, test ordering based on case history and sample pre-treatment), analytical (e.g., virology [14], chemistry [2], bacteriology [14], or histopathology [15] testing), or post-analytical (e.g., data transcription, interpretation of results, and reporting) phases. However, those PTs do not cover the entire (i.e., end-to-end) investigative process. Some organizations evaluate multiple components of the diagnostic investigation process in their internal exercises as part of routine certification, licensing, or training programs, which are mostly related to infectious diseases. For example, participants may be tasked with identifying bacteria and/or viruses in specimens from an ill animal using various diagnostic kits.

Evaluations of a toxicosis investigation are more challenging because the chemistry component requires expensive analytical instrumentation. Based on personal communication, some certification and training pathology and toxicology programs conduct exercises in which participants are provided with (rather than generating) chemistry results, along with clinical and pathological data, for a comprehensive evaluation of toxicosis causes. Unfortunately, to our knowledge, the design details and outcomes of those exercises are not publicly available.

One recently reported PT [16] resembles the diagnostic PT described to a certain extent by evaluating the entire end-to-end diagnostic testing which includes pre-analytical, analytical, and post-analytical phases using the integrated approach [17]. However, it differs from the diagnostic PT described in key aspects, such as focusing on human cancer biomarker testing rather than investigating animal exposure to an unknown toxicant.

### 4.2 Significance of the diagnostic PT design used

The design of this diagnostic PT allows for the evaluation of not only the analytical component (i.e., chemistry analysis) of the diagnostic investigation but also the entire investigative process as a holistic, step-by-step procedure using an integrative, multidisciplinary approach. Importantly, the conditions of this diagnostic PT closely mimic a typical animal toxicosis case, where investigators must first determine which chemistry analyses to perform, then accurately test the samples, and finally interpret the diagnostic significance of the analytical findings.

Moreover, the assessment of the chemistry component (i.e., analytical testing of samples) in this diagnostic PT (Fig. 2) provides a higher degree of confidence, as it is less prone to bias compared to a typical chemistry PT (Fig. 1). In a typical chemistry PT, participants are informed in advance about which analytes they need to target and which methods they will use to analyze blind-coded samples. This prior knowledge makes the analytical part less challenging for participants, as it gives chemists an unfair advantage by allowing them to better prepare for testing. Specifically, prior to participating in a PT, when the analyte is known, a participant can optimize and clean analytical instruments, prepare fresh analytical standards, perform specific cleaning of glassware and equipment like homogenizers and others. In contrast, the conditions of this diagnostic PT (Fig. 2) closely simulate real-world analytical testing, where laboratories test samples under standard conditions without extra preparation, making this PT a more authentic and unbiased assessment of analytical chemistry capabilities.

### 4.3 Limitations and challenges of the diagnostic PT conducted

Although the PT evaluated the entire end-to-end diagnostic investigation as a whole process, certain specific parts, such as sample collection, receipt, storage, and pre-treatment, were omitted from the detailed assessment. It is important to note that the brain and liver samples were not from a real case (i.e., the unknown liver was prepared by fortification with lead) and were pre-homogenized by the organizers. Additionally, the histology image was provided by the organizers rather than generated by the participants.

In this PT, participants were given freedom in the format of their histopathology reports, resulting in high variability in depth and clarity. This variability could potentially have a significant impact in real cases and may need to be addressed in future PTs [16].

PT organizers did not establish the concentrations of any other analytes in the PT samples (i.e., it was out of scope) rather than lead. This prevented the organizers from quantitatively evaluating the accuracy of results for other analytes (e.g., sodium and sulfur) submitted by participants. For example, Lab ID #10 reported brain sodium at 2240 µg/g, which was higher than what the organizers expected, considering that the brain was taken from a healthy animal (often <2000 µg/g). The true sodium concentration was not established by the organizers. While this result did not affect the participant’s diagnosis of lead toxicosis, it may not be accurate from an analytical chemistry perspective and could impact diagnosis in real cases, as this concentration (2240 µg/g) is near the margin of diagnostically significant values.

PT organizers did not establish the concentration of lead in the brain sample because the brain is considered an unsuitable tissue for lead toxicosis testing due to its limited accumulation of lead. Lab ID #12 reported 1.06 µg/g of lead in the brain (Table 3), which was higher than what the organizers expected, considering that the brain was taken from a healthy animal. While this result did not affect the participant’s reported diagnosis (lead toxicosis), it may be inaccurate (i.e., potentially a false positive) from an analytical chemistry perspective, particularly if the method is intended for research purposes, where standards are generally more stringent than those for diagnostic purposes (i.e., focusing on values close to diagnostic concern or action levels). Since the true lead concentration in the brain was not established by the organizers (i.e., it was out of scope), the participant was not evaluated for this result.

Overall, organizing this diagnostic PT was more demanding than a typical chemistry PT. For example, the organizers needed to develop a case that was neither too easy nor too difficult to diagnose and had to ensure that the chemical analysis did not require rare analytical instrumentation. However, this PT was similar to typical PTs regarding other resources, such as sample preparation, stability and homogeneity verification, shipping, and evaluation of results according to ISO standards [12].

### 4.4 Comparison of the diagnostic PT to typical chemistry and other PT types

In both typical chemistry PTs (Fig. 1) and the diagnostic PT (Fig. 2), participants are unaware of the sample content. However, they are aware that their performance is being evaluated, which classifies these PTs as "declared" or "open" (as opposed to "blind") according to terminology of some medical [18] and forensic [19] PT programs. In blind PTs, participants are unaware that they are being evaluated. Blind PTs are generally considered the most suitable for evaluating laboratory performance because they fully mimic real-world testing conditions. Blind PT samples are processed as part of the routine lab workflow, thereby addressing some of the key issues found in declared chemistry PTs, such as:

(a) *The averaging problem*: This occurs when a PT participating laboratory analyzes a test sample multiple times and reports the average value [20] whereas, during routine analysis of real-world samples, they typically analyze each sample only once. This averaging masks variability and outliers, undermining the assessment of accuracy and precision.

(b) *The Hawthorne effect* [21]: This refers to the tendency for people to change their behavior when they know they are being evaluated. This can significantly inflate performance results. Anecdotal evidence suggests that some laboratories temporarily halt non-PT activities to focus solely on PT samples by recalibrating analytical instruments, balances and pipettes; cleaning equipment (e.g., homogenizers and glassware); preparing fresh analytical standards; and re-training personnel on the specific SOP used for the PT.

(c) *Inadequate blinding of participants regarding PT samples*: Poor PT organization can allow participants “to know too much” about test samples composition or the PT design-scheme. Specifically, in quantitative chemistry PTs participants should not be aware of all three parameters: (i) the number of analyte levels (for example, test samples fortified with analyte at, below and above the concern level means there are three levels), (ii) the specific analyte concentration in each level and (iii) the number of replicates used at each level. If participants know either (i) or (iii), they could group similar results and identify potential outliers, even without knowing the exact analyte concentrations [22]. In addition, (i) and (iii) should vary randomly in re-occurring PTs.

Overall, despite the advantages of blind PTs [18], they are complex, resource-intensive and challenging to organize [23]. Additionally, many laboratories are reluctant to participate in them [24]. The diagnostic PT outlined here, while not classified as blind according to some PT programs, better addresses the issues (see a, b and c above) of typical chemistry PTs. The design of this diagnostic PT represents a step forward in providing unbiased performance assessments for VDLs and should therefore receive consideration from PT providers and accreditation bodies in the future.

## CRediT authorship contribution statement

**Fritz Scott:** Writing – review & editing, Validation, Methodology, Data curation, Conceptualization. **Nemser Sarah M.:** Writing – review & editing, Validation, Project administration, Methodology, Formal analysis, Data curation, Conceptualization. **Chen Yang:** Writing – review & editing, Validation, Methodology, Formal analysis, Conceptualization. **Petrey Marissa:** Writing – review & editing, Validation, Methodology, Formal analysis. **Reddy Ravinder:** Writing – review & editing, Validation, Supervision, Project administration, Methodology, Formal analysis, Data curation, Conceptualization. **Tyson Gregory:** Writing – review & editing, Supervision, Methodology, Formal analysis, Conceptualization. **Tkachenko Andriy:** Writing – original draft, Validation, Methodology, Formal analysis, Data curation, Conceptualization. **Dark Michael:** Writing – review & editing, Methodology, Formal analysis, Conceptualization. **Kmet Matthew:** Writing – review & editing, Project administration, Methodology, Formal analysis, Data curation, Conceptualization. **Miller Megan R.:** Writing – review & editing, Validation, Methodology, Formal analysis, Data curation, Conceptualization. **Williams Bruce:** Writing – review & editing, Methodology, Formal analysis, Conceptualization. **Walsh Tim:** Writing – review & editing, Methodology, Formal analysis, Conceptualization. **Rotstein David:** Writing – review & editing, Validation, Methodology, Conceptualization.

## Declaration of Competing Interest

The authors declare that they have no known competing financial interests or personal relationships that could have appeared to influence the work reported in this paper.

## Data Availability

The data that has been used is confidential.
